# Design of an Effective Prosthetic Hand System for Adaptive Grasping with the Control of Myoelectric Pattern Recognition Approach

**DOI:** 10.3390/mi13020219

**Published:** 2022-01-29

**Authors:** Yanchao Wang, Ye Tian, Haotian She, Yinlai Jiang, Hiroshi Yokoi, Yunhui Liu

**Affiliations:** 1 School of Mechatronical Engineering, Beijing Institute of Technology, Beijing 100081, China; 3120170102@bit.edu.cn (Y.W.); 3120150094@bit.edu.cn (H.S.); yunhui.liu@gmail.com (Y.L.); 2 Beijing Advanced Innovation Center for Intelligent Robot and System, Beijing 100081, China; jiang.yinlai@uec.ac.jp (Y.J.); yokoi@mce.uec.ac.jp (H.Y.); 3 Graduate School of Informatics and Engineering, The University of Electro-Communications, Tokyo 1828585, Japan; 4 Department of Mechanical and Automation Engineering, The Chinese University of Hong Kong, Hong Kong 999077, China

**Keywords:** prosthetic bionic hand, sEMG signal, LDA, motion recognition

## Abstract

In this paper, we develop a prosthetic bionic hand system to realize adaptive gripping with two closed-loop control loops by using a linear discriminant analysis algorithm (LDA). The prosthetic hand contains five fingers and each finger is driven by a linear servo motor. When grasping objects, four fingers except the thumb would adjust automatically and bend with an appropriate gesture, while the thumb is stretched and bent by the linear servo motor. Since the change of the surface electromechanical signal (sEMG) occurs before human movement, the recognition of sEMG signal with LDA algorithm can help to obtain people’s action intention in advance, and then timely send control instructions to assist people to grasp. For activity intention recognition, we extract three features, Variance (VAR), Root Mean Square (RMS) and Minimum (MIN) for recognition. As the results show, it can achieve an average accuracy of 96.59%. This helps our system perform well for disabilities to grasp objects of different sizes and shapes adaptively. Finally, a test of the people with disabilities grasping 15 objects of different sizes and shapes was carried out and achieved good experimental results.

## 1. Introduction

As an important part for individuals with upper-limb differences, a prosthetic bionic hand is indispensable to keep the structural integrity of the human body. For the study of prosthetic bionic hands, many studies contribute greatly. For example, Dr Paul Chappell from the University of Southampton in the UK developed the Southampton Hand [1] series based on the observation of human hands and fingers when grasping. In this scheme, each finger has three joints except the thumb, and the motion of two joints at the end are correlatively coupled. However, the hand only has three fingers, which is too small for people to act like ordinary people. Dalley et al. [2] developed a hand with 16 joints driven by five independent actuators, which can provide eight hand postures. However, the specific control process is too complicated, which is not practical for individuals with upper-limb difference. Another study is the i-Limb hand, which has five independently controlled fingers that is controlled by action signals from two electrodes [3]. There are also some other classic prosthetic bionic hands, including Okada hand [4], Stanford/JPL [5], Shadow hand [6], Utah/MIThand [7,8,9], Hitachi hand [10], etc. In all, the research on intelligent prosthetic hands is relatively too complicated, resulting in various inconveniences for the individuals with upper-limb difference in practical application. However, due to seeking more freedom, the motor driving system of these prosthetic hands are relatively too complicated in hardware structure and control system, leading to the weak strengthen of hand structure and poor real-time performance. Therefore, improving prosthesis control is a key requirement for enhancing prosthesis satisfaction. By improving intuitive control prosthesis, users may experience decrease cognitive effort and decrease dependency on vision to guide and monitor actions.

Among them, the Ottobock hand [11] produced by Otto Bock Company in Germany is one of the most successful and widely used prosthetic hands in the world. Although this kind of hand can achieve good accuracy, its control movement is fixed, which could not realize the adaptive grasp of objects of different shapes. In order to realize the adaptive grasping function of the prosthetic bionic hand, many studies begin to concentrate on the feature extraction method and advanced intelligent algorithms.

The surface electromechanical signal (sEMG) has a peak value of 0–6 mv and a frequency range of 0–500 hz is one-dimensional time-series signal recorded by electrodes on the surface of the muscle during neuromuscular system activity [12,13] have the properties of non-stationary, non-linear, complexity, and large variation. At the same time, when the handicapped wear the prosthetic bionic hand, there will also be sweating of the skin, muscle fatigue [14,15,16,17], ECG noise, device noise [18,19] and other environmental conditions. These characteristics make it so difficult to analyze surface electromechanical signals under multiple action modes well. Generally, the recognition of human movement intention through electromyographic signals is mainly divided into three steps [20]: signal preprocessing, feature extraction and feature classification [21,22,23]. The signal preprocessing process generally includes signal amplification, filtering and notch. This process is a significant step, which directly affects the reliability of signals [24]. Feature extraction is used for extracting relevant information from sEMG signal, and rejecting noise and other unimportant components. Feature extraction of sEMG signals mainly includes time domain analysis, frequency domain analysis and time-frequency domain analysis. Time domain analysis includes Integrated EMG, Mean Absolute Value (MAV), Root Mean Square (RMS), Zero Crossing, etc. [25]. Frequency domain analysis includes Autoregressive coefficient (AR), Modified Median Frequency (MMDF), etc. [26], and time-frequency domain analysis includes Wavelet Transform (WT), Wigner–Ville distribution (WVD), etc. [27].

Among the feature extraction methods, time domain analysis is computational simplicity and effective, which is widely used in many studies. For example, Hudgins et al. recognized four forearm motions by using five time domain features including MAV, Mean Absolute Value Slope (MAVS), Zero crossing (ZC), Slope Sign Changes (SSC) and Waveform Length (WL), and gained an average accuracy rate of 91% [28]. Kim et al. successfully classified four wrist movements by using Integrated Absolute Value (IAV) and Root Mean Square (RMS) [29].

For sEMG-based control of prosthetic hands, there are many classification algorithms, such as linear discriminant analysis (LDA) [30], fuzzy logic, artificial neural networks (ANN), and support vector machine (SVM) [31]. For example, S.M. Mane [32] used the ANN algorithm for hand gesture recognition; H. Zhang [33] adopted LDA for sEMG pattern recognition; K. Xing [34] applied the support vector machine (SVM) into the real-time sEMG pattern recognition system for the control of the prosthetic hand. However, for the control of prosthetic hands, we need to consider two indicators, running speed and recognition accuracy.

To select a suitable algorithm for the control of prosthetic hand, Sumit A [35] identify different hand movements sEMG signals of prosthesis hand with LDA, SVM and ANN algorithm for comparison, and proved that the LDA algorithm can achieve a high accuracy, as well as faster running behavior than other algorithms. Besides, D. Zhang [36] also claimed that LDA can achieve better control performance in EMG classification for a prosthetic hand. The same work can also be seen in the work from C. Antuvan [37]. Therefore, we can conclude that the LDA algorithm can perform better in the classification work in sEMG signals for the control of prosthesis hand, whether in recognition accuracy or in running speed. So, we choose LDA algorithms for intension recognition in our work.

Based on the analysis above, this paper designed an adaptive prosthetic bionic hand with an LDA algorithm. We present the mechanical structure design of our prosthetic bionic hand and also analyze the movement track of the finger. Then the control strategy is designed based on the sEMG signal. In the control process, we firstly collect the sEMG data and segment it with a sliding window for feature extraction. Then the LDA algorithm is applied to recognize the motion intension through these features. Once the algorithm recognizes a certain movement, the system will send the command to the motor of BIT hand C so as to realize the grasping and opening action.

The contribution of this study can be summarized as four aspects: (1) The bionic hand adopts the connecting rod drive design so that the bionic hand can better adapt to the grasping action of objects of different shapes and sizes. This kind of design is more anthropomorphic than the Ottobock bionic hand on the market. (2) In the grasp control strategy of the bionic hand, we adopt two closed-loop control strategies of the current loop and position loop, which makes the control of the bionic hand safer and more reliable. (3) In order to make the hand light, cheap and durable as much as possible, we design it with aluminum alloy, nylon and rubber, which is only 332 g. This kind of design can help people grasp objects more freely than other designs. (4) We adopt LDA linear classifier for the signal detection from the people, which makes the control process faster and more accurate.

## 2. Method

### 2.1. System Components of Hardware System

The prosthetic bionic hand designed in this paper is named BIT Hand C. In order to make the hand as light and convenient as possible, we design it with aluminum alloy, nylon and rubber. The total mass of BIT Hand C is only 332 g. It is driven by five electric motors, which has the characteristics of small size, high accuracy and self-locking. Four linear actuators (LA10-21D) control the fingers and one linear actuator (LAS10-23D) control the thumb. The linear servo driver, produced by Beijing Inspire Robots Technology Co., Ltd., adopts DC brushed micro motor. Its reducer adopts a two-stage planetary gear reduction device to form a large reduction ratio and realizes low speed and large torque.

**Figure 1:**
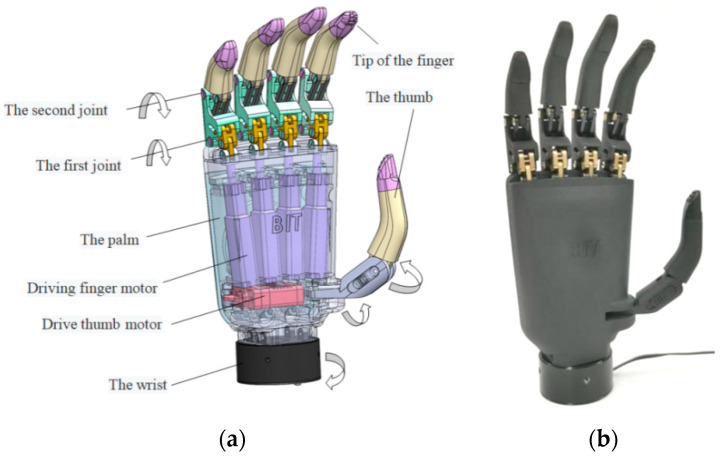
The prosthetic bionic hand. (**a**) 3D modeling of BIT Hand C. (**b**) Photograph of BIT Hand C.

The BIT Hand C developed in our study is shown in Figure 1. It has 5 fingers with a total of 11 joints and 6 degrees of freedom. Each finger has its own independent motor drive so that each finger can realize independent movement. The micro linear servo drives of this prosthetic bionic hand are all placed inside the palm. Placing all actuators inside the palm makes the design simpler and control more efficient. On the premise of great power, the weight of the whole prosthetic bionic hand is also light, which is acceptable to individuals with upper-limb differences. The hand, BIT Hand C, has a strong grasping ability, it can lift up to 5 KG of weight, and the maximum pinching force of the fingertips is up to 12 N.

BIT Hand C has the function of wrist rotation, but the wrist rotation movement is passive, and there is no rotating motor. The wrist is controlled by the damping force of the rubber apron in the process of rotating motion. The damping force setting of wrist motion is realized by adjusting the fastening force of bolts. A slip ring mechanism is arranged in the structure of the wrist so that the wrist movement of the bionic hand will not cause wear and damage to the connection power line of the motor inside the bionic hand palm.

**Figure 2:**
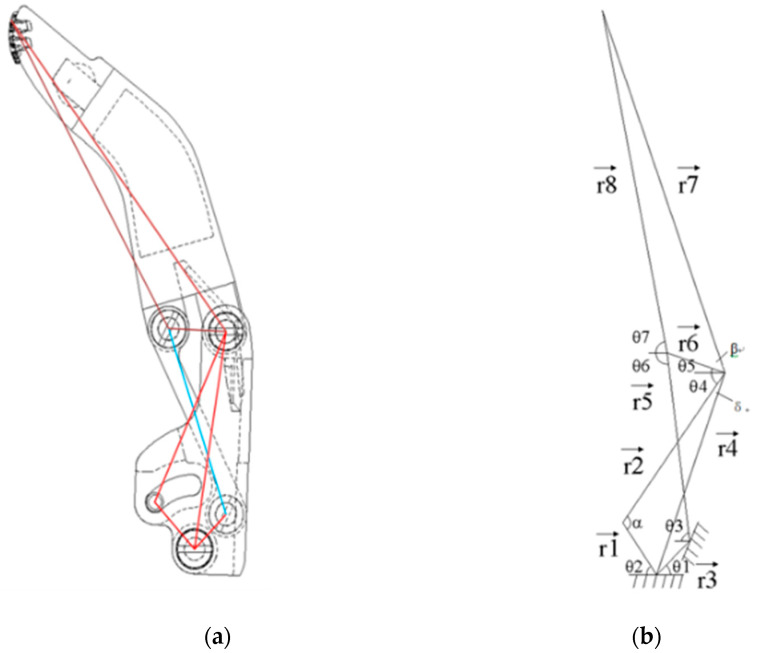
Design of connecting rod of finger mechanism. (**a**) The connecting rod structure of the finger, (**b**) Finger connecting rod structure schematic diagram.

In order to make the hand movement simpler and more effective, each finger is designed with one less joint, namely one degree of freedom. The finger trajectory analysis model is composed of three links, which can be calculated by vector equation, as shown in Figure 2a. The vector diagram of the finger kinematics is presented in Figure 2b.

The closed vector equation of the finger linkage shown in Figure 2b can be written as:

(1) $$\vec{r_{4}}+\vec{r_{6}}=\vec{r_{3}}+\vec{r_{5}}$$

(2) $$\vec{r_{1}}+\vec{r_{2}}+\vec{r_{7}}=\vec{r_{3}}+\vec{r_{5}}+\vec{r_{8}}$$

With XY components for each closed vector equation, the two vector Equations (1) and (2) can rewrite with four XY scalar component equations:

(3) $$r_{4}{\sin\theta }_{4a}+r_{6}\sin\theta_{5}=r_{3}{\sin\theta }_{1}+r_{5}{\sin\theta }_{3};$$

(4) $$r_{4}{\cos\theta }_{4a}-r_{6}\cos\theta_{5}=r_{3}{\cos\theta }_{1}-r_{5}{\cos\theta }_{3}$$

(5) $$r_{1}{\sin\theta }_{2}+r_{2}\sin\theta_{4}+r_{7}\sin\theta_{5a}=r_{3}{\sin\theta }_{1}+r_{5}{\sin\theta }_{3}+r_{8}{\sin\theta }_{7};$$

(6) $$r_{1}{\cos\theta }_{2}-r_{2}\cos\theta_{4}+r_{7}\cos\theta_{5a}=-r_{3}{\cos\theta }_{1}+r_{5}{\sin\theta }_{3}+r_{8}{\sin\theta }_{7}$$

where $\theta_{4a}=\theta_{4}+\delta$ and $\theta_{5a}=\theta_{5}+\beta$, δ and β are constants.

**Figure 3:**
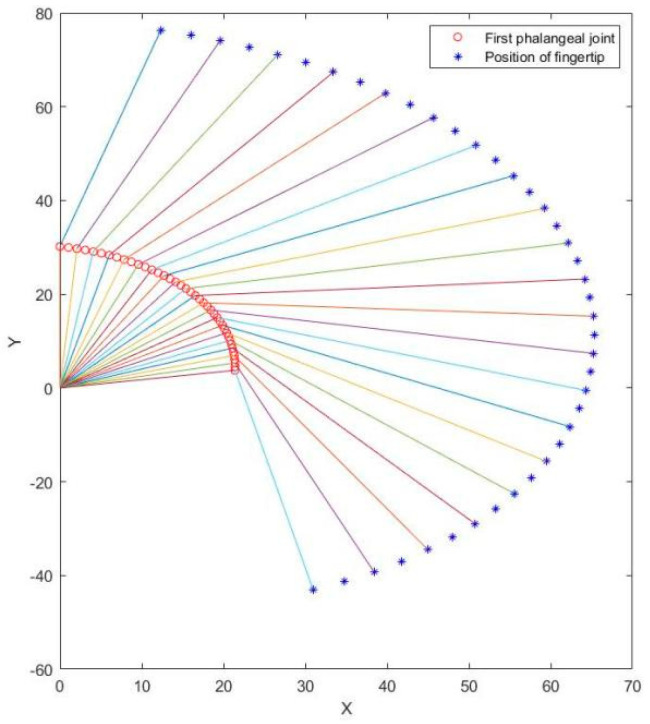
Design of connecting rod of finger mechanism.

Then the system can be solved with these equations by using MATLAB for finger position analysis. Figure 3 shows the finger movement trajectories of BIT Hand C in different postures and manipulable space. As can be seen from the figure, the maximum movement angle of the first knuckle can reach 84.5 degrees, and the maximum movement angle of the second knuckle is 135 degrees. It can be seen that BIT Hand C can fit the maneuverability space of the normal hand well. While the finger of the Attobock hand is only composed of a solid structure, and the grasping finger has only one degree of freedom, when the hand grasps an object, the contact surface between the finger and the object mostly has only one fulcrum. Furthermore, BIT Hand adopts an underactuated connecting rod mechanism design, which not only ensures the driving motor pulling force but also enables fingers to realize underactuated grasping ability, so as to ensure at least two surfaces contact between fingers and objects, which can better ensure the stability of grasping action.

**Figure 4:**
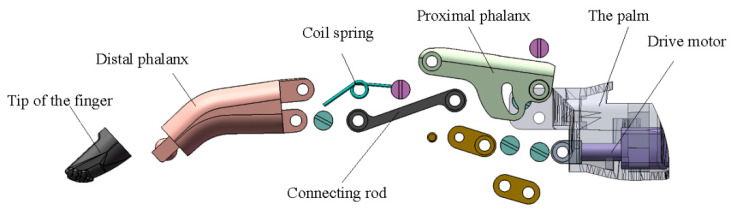
The exploded view of the finger.

The four fingers of BIT Hand C are driven by the connecting rod mechanism, which is designed to suit the driving principle. Take the right index finger, for example, we describe the finger structure in detail, as shown in Figure 4.

The finger is mainly composed of the distal phalanx, proximal phalanx, fingertip, connecting rod, coil spring and pin. Nylon is selected for distal phalanx and proximal phalanx, making it lightweight. Fingertip is made of rubber for easier grasping for different objects. The connecting rod is made of aluminum alloy, making it strong.

The finger of BIT Hand C is a kind of compliant linkage mechanism. It only needs a micro linear private server driver (LA10-21D) to complete the basic movements of grasping. Its maximum push force is 70 N, and the maximum stroke of these two drives is 10 mm. When the push rod of the drive moves to the right, the finger will be bent under the pull of the link. The extension spring will bend to a certain extent, showing a relatively large displacement without any permanent deformation or torsion. The extension spring is arranged at the second knuckle joint of the finger. The bionic hand has a fingertip grip of 12 N.

**Figure 5:**
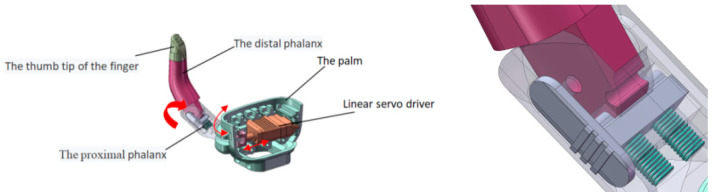
The design of the thumb.

The thumb part, the maximum angle of rotation of which is 75 degrees consists of the proximal knuckle, distal knuckle, and fingertip, as shown in Figure 5. A linear servo drive placed in the palm of the hand enables it to rotate. However, the opening and closing movement of the thumb is passive, which requires individuals with upper-limb differences to adjust the motion position through their own healthy hands. A linear servo drive is LAS10-023D, with a maximum push force of 105 N, which is used for rotation drive.

### 2.2. Control Strategy for Prosthetic Bionic Hand

#### 2.2.1. Control Process of BIT Hand C

**Figure 6:**
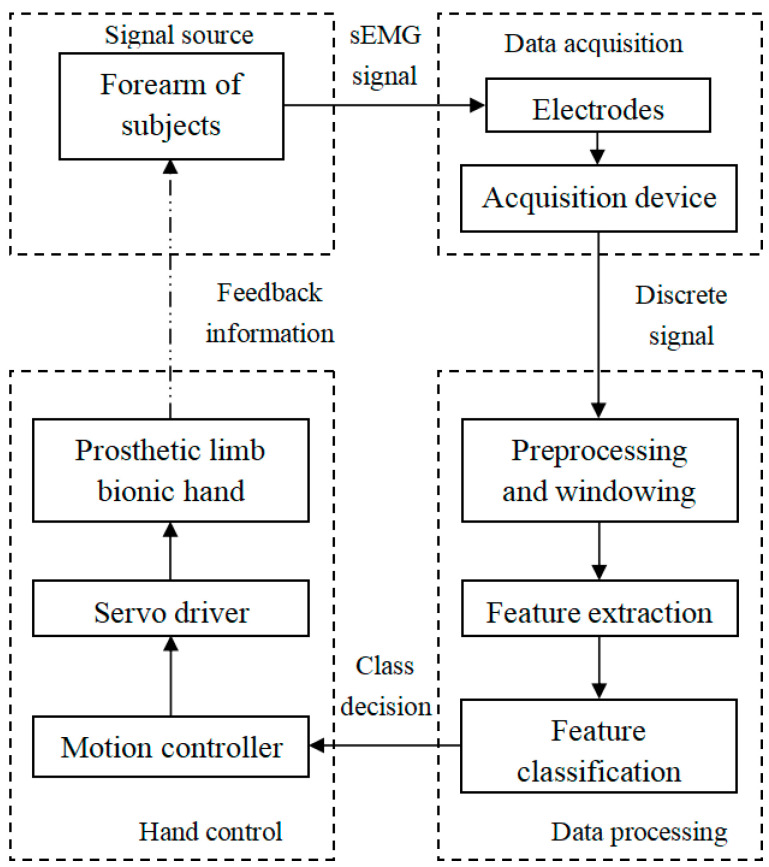
Control flow chart of BIT hand C.

The BIT hand C is controlled by surface electromechanical signals(sEMG). The control system is mainly divided into four parts, including signal source (EMG signal from the forearm), data collection, data processing, and decision and control. Figure 6 shows the flow of the control system. The sEMG signals are collected from 2 channel electrodes. Data processing includes signal preprocessing, window processing, feature extraction and feature classification. The final classification results are sent to the control system, which controls the action of the prosthetic bionic hand through the actuator.

#### 2.2.2. Control Strategy of BIT Hand C

**Figure 7:**
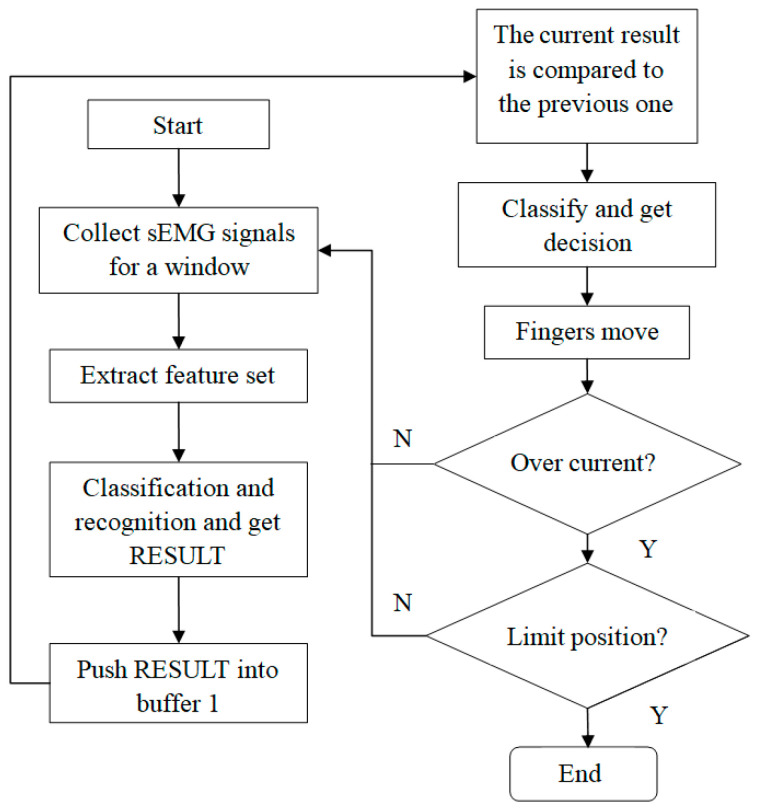
The working diagram of the whole control system.

The control strategy of BIT hand C is shown in Figure 7. Firstly, the sliding window is applied to process real-time data into frames, and the window size and frameshift are 250 ms and 100 ms, respectively. After data amplification and filtering, feature extraction and action pattern recognition are carried out on the processed data, then the final classification results are imported into the control system. Next, we adopt a classification algorithm to classify the mode of it, that of grasping or opening. Once the algorithm detects the mode successfully, it can be then be judged as corresponding motion. Finally, the system will send the command to the motor of BIT hand C and then it will be driven to move, so as to realize the grasping and opening action.

The highlight of this study is that our control system sets up two closed-loop controls, the current loop and the position loop to ensure the adaptively grasping of objects of different shapes and sizes. When the BIT hand C is working, the system will monitor the current flow through each motor. Once the current flow is recognized as a certain intention, the motor will be pushed to grasp or open. In this way, the adaptive grasp of BIT hand C can be realized, which can better control various daily grasp movements of individuals with upper-limb differences.

## 3. Data Processing

### 3.1. Data Collection

The prosthetic bionic hand designed is an adaptive under-actuated prosthetic hand, which only needs two electrode channels sEMG signal for high control accuracy. In this way, it only needs little calculation work for data processing and thus improves the running speed to make sure the real-time performance.

**Figure 8:**
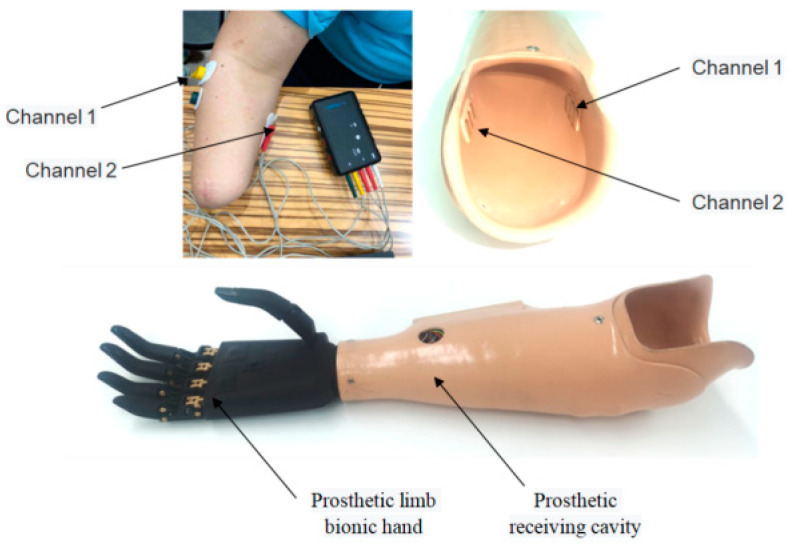
The position of two channel electrodes.

**Figure 9:**
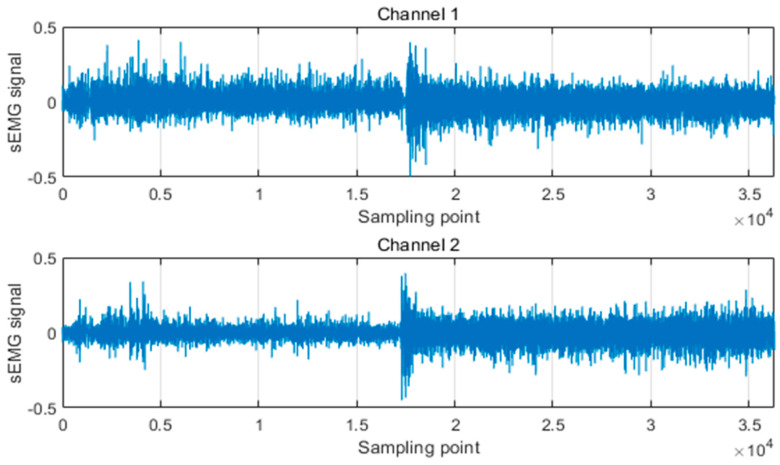
SEMG signals from two electrodes.

Before electrodes are placed, the skin paste should be treated, including removing hair, removing sweat, and wiping clean with alcohol. As experiment shows, we select brachioradialis muscle and extensor digitalis muscle as objects to obtain the sEMG signals, which show obvious change during different hand movements. The placement position of the electrodes is shown in Figure 8. In this paper, we set the sampling frequency of the surface electromechanical signal at 1000 Hz. The amplitude of the original EMG signal is only 0–6 Mv, so we design the amplifier circuit to amplify it, and we also perform a filter to remove the noise. Finally, the usable EMG signal can be obtained. In Figure 9, we present the raw sEMG signal from two electrodes.

### 3.2. Feature Extraction

**Figure 10:**
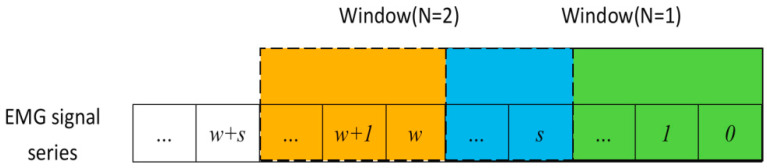
Sliding window scheme.

In the feature extraction process, we adopt the sliding window to segment sEMG signals. The detail of the method is shown in Figure 10. To make sure real-time detection and decision, the sliding window needs to be overlapped. It is known that the higher the overlapping radio is, the faster response speed it has. However, it is not to see that we should set it as large as possible without limitation. We should also take recognition and decision accuracy into consideration. Additionally, a suitable size of sliding window can not only improve the algorithm speed but also improve the recognition accuracy. In this paper, we set the window length at 250 ms and the increment at 100 ms for better performance.

In this paper, we adopt time domain information for feature extraction. Among them, the integral myoelectric value (iEMG), denoted in Equation (7), the root mean square value (RMS), denoted in Equation (8), and mean absolute value (MAV), denoted in Equation (9), can reflect the variation of sEMG signal amplitude.

(7) $$iEMG=\int_{t}^{t+T}\left|EMG\left(t\right)\right|dt$$

(8) $$RMS=\sqrt{\frac{1}{N}\sum_{n=1}^{N}x_{n}{}^{2}}$$

(9) $$MAV=\frac{1}{N}\sum_{n=1}^{N}\left|x_{n}\right|$$

where *N* is the number of samples in a certain window, and *x_n_* is the *n*th sample in the *EMG* signal.

Zero crossing (ZC) is the frequency sEMG signals that cross the x-axis. In order to avoid the influence of low voltage fluctuation and background noise, a threshold is applied to the signal amplitude, as shown in the following formula:

(10) $$ZC=\sum_{i=1}^{N-1}\left[sgn\left(x_{n}\times x_{n-1}\right)\cap^{\;}\left|x_{n}-x_{n-1}\right|\geq threshold\right];sgn\left(x\right)=\{\begin{array}{c} 1,\;if\;x\;\geq threshold\\ 0,\;\;otherwise \end{array}.$$

Variance (Var) is a measure of dispersion degree for a set of data. The equation is shown below:

(11) $$Var=\frac{\sum_{i=1}^{n}{\left(X_{i}-\;\bar{X}\right)}^{2}}{n-1}$$

where X_i_ represents the *i*th sample and X represents the mean value of sample data, and n represents the total number of samples.

### 3.3. Feature Classification

There are many algorithms for feature classification, which are mature and commonly used at present, such as Linear Discrimination Analysis (LDA), Support Vector Machine (SVM), K-Nearest Neighbor (KNN), Artificial Neural Network (ANN), decision tree (DT) and so on. Linear discriminant analysis is a simple but effective method for classification, which can achieve fast recognition. The basic idea of *LDA* classifier is to find an optimal discriminant vector space to maximize the ratio of the inter-class dispersion and intra-class dispersion of samples projected.

The calculation steps of the LDA algorithm are shown as follows:

(a) Firstly, calculate the mean vector of each class using Equation (12):
  (12) $$u_{i}=\frac{1}{n_{i}}\sum_{x\in class_{i}}x$$
  where *n_i_* is the number of samples of each class, and *x* denotes the original feature vectors of each class.
(b) Secondly, calculate the mean of samples using Equation (13):
  (13) $$u=\frac{1}{m}\sum_{i=1}^{m}x_{i}$$
  where *m* is the total number of all classes.
(c) Thirdly, calculated the inter-class divergence matrix and the in-class divergence matrix using Equations (14) and (15).
  (14) $$S_{b}=\sum_{i=1}^{c}n_{i}\left(u_{i}-u\right){\left(u_{i}-u\right)}^{T}$$
  (15) $$S_{\omega }=\sum_{i=1}^{c}\sum_{x\in class_{i}}\left(u_{i}-x_{k}\right){\left(u_{i}-x_{k}\right)}^{T}$$
  Here, it is important to note that a weighted average is needed when calculating the total $S_{b}$ and $S_{\omega }$, because the number of samples for each class may be different.
(d) Finally, we can express the Fisher criterion in terms of $S_{b}$ and $S_{\omega }$ as:
  (16) $$J\left(\omega \right)=\frac{\omega^{T}S_{b}\omega }{\omega^{T}S_{\omega }\omega }$$

By maximizing the generalized Rayleigh quotient, we use the Lagrange multiplier method to find the required eigenvectors. Then the original feature matrix can be projected by:

(17) $$y=\omega^{T}x$$

The matrix y represents the projection eigenvector of original data, through which we can obtain the best recognition accuracy.

## 4. Experiments Results

### 4.1. Intention Recognition Results

We, in total, asked 10 subjects to conduct the experiment, of which 9 subjects were able-bodied, between 28 and 38 years old, and one individual with upper-limb difference, aged 52. All subjects provided informed consent before the experiment. The experimental procedures were reviewed and approved by the Ethics Committee of The University of Electro-Communications (No. 10006(5)).

**Figure 11:**
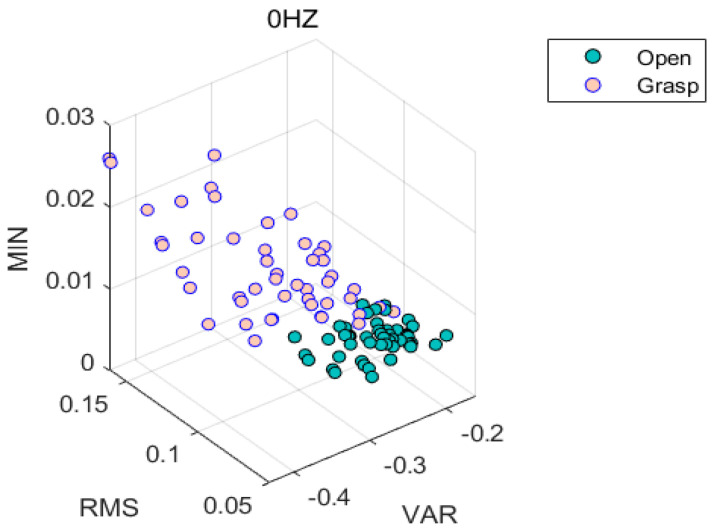
Opening and gripping action recognition with LDA algorithm.

For the classification of LDA classifier, we finally collected 128 samples for each subject. Then, 64 were randomly selected for training, and the other 64 samples were used for testing. The subjects were required to perform hand opening and grasping movements, and each movement hold for 5 s. Every hand gesture was repeated 10 times. To avoid muscle fatigue, the subjects were given a five-minute break before the next test. Then, VAR, RMS and MIN features were extracted and the LDA algorithm was applied for action recognition, as shown in Figure 11.

Green points represent the eigenvalue when the hand is open, while pink points represent the eigenvalue when the hand is grasped. It can be seen from Figure 11 that the three features, VAR, RMS, MIN, can be well distinguished using the LDA classification algorithm. After training, we tested the new action data and calculated its accuracy with Equation (18).

(18) $$Accuracy=\frac{NO.\;of\;all\;the\;features-NO.\;of\;feature\;classification\;errors}{NO.\;of\;all\;the\;features}\times 100\%$$

As the results show, we can achieve an average accuracy of 96.59% with the LDA algorithm.

To prove the effectiveness of the LDA algorithm, we compare the recognition accuracy with other recognition algorithms, such as native bayes (NB), support vector machine (SVM), decision tree (DT), nearest neighbor (KNN) and linear discriminant analysis (LDA), as shown in Table 1. For convenience, we only present the results of six subjects.

**Figure 12:**
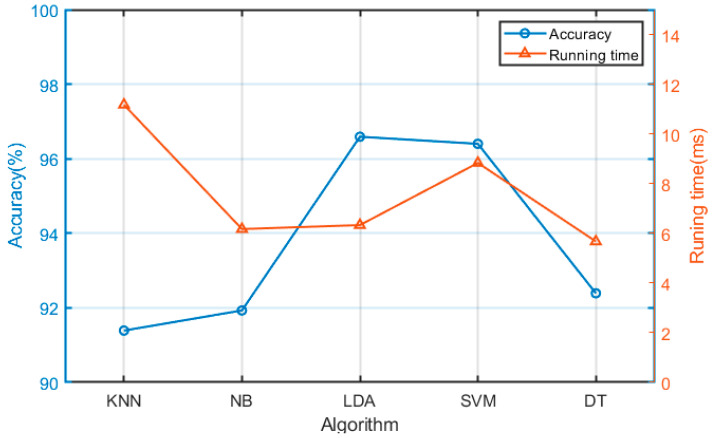
Comparison of different classifiers in terms of accuracy and running time.

Among the algorithms, LDA shows high accuracy. Furthermore, we compare the computation time of different classifiers, as shown in Figure 12.

From Figure 12, we can see that LDA can achieve an accuracy of 96.59%, which is greatly higher by 4.2% and 4.66% than that of DT and NB respectively. Furthermore, for running speed, LDA only needs 6.33 ms, which is only slightly slower by 0.5 ms and 0.16 ms than that of DT and NB respectively. Weighing these factors, we conclude that LDA performs better than other algorithms.

**Figure 13:**
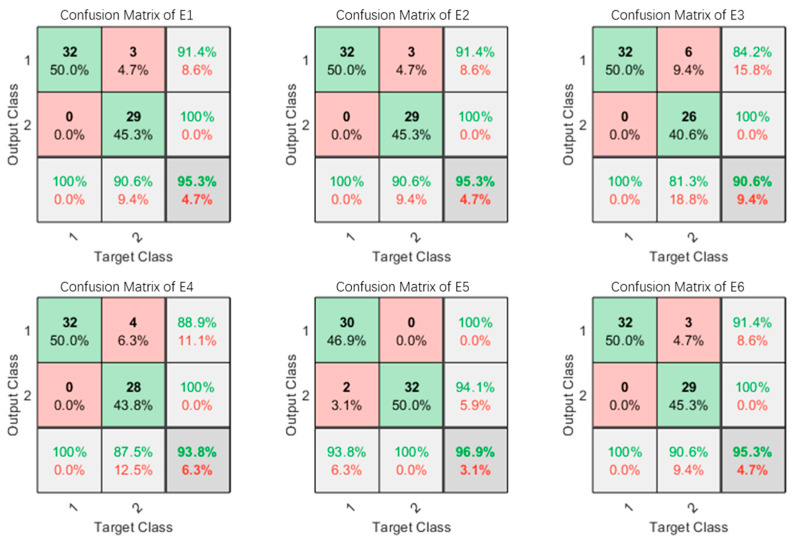
Confusion matrix of subject 1 for certain 6 times experiments (E1–E6). 1—grasping, 2—opening.

For the observation of the classification results of the LDA algorithm clearly, we present the confusion matrix of subject 1 for certain 6 times experiments, for example, as shown in Figure 13.

### 4.2. Grasp Test Results

**Figure 14:**
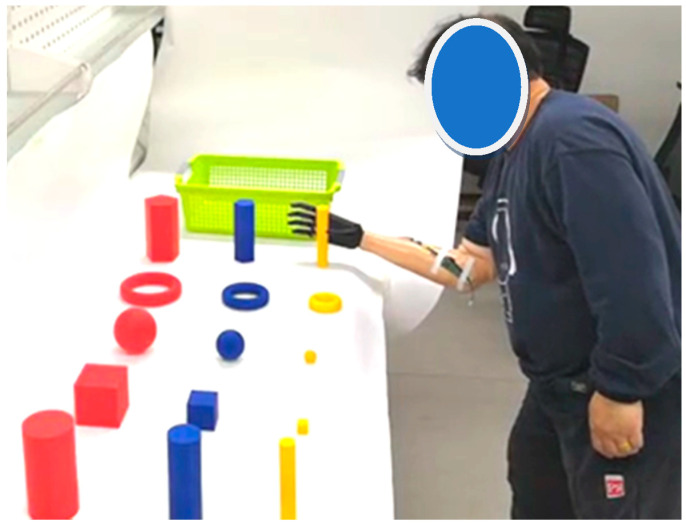
Tests on individuals with upper-limb difference grasping different objects.

In this part, we verify the reliability of the control strategy based on the LDA algorithm. During tests, subjects are asked to rest for five minutes instead of conducting five grip tests to avoid muscle fatigue. The test content is to control BIT hand C to grasp objects of different shapes and sizes. In this experiment, five kinds of objects with different shapes were selected, including cylinder, cube, sphere, torus and pentagonal column. Each shape comes in three sizes: large, medium and small, as shown in Figure 14.

**Figure 15:**
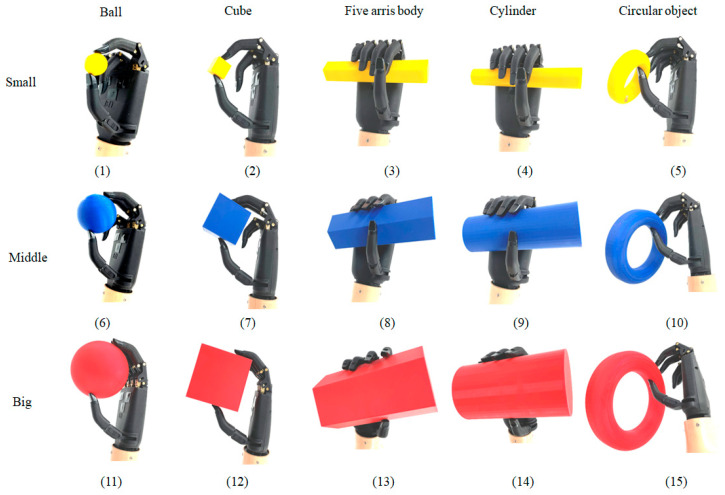
Grasp test of different objects of BIT hand C.

Figure 15 shows the hand posture for grasping objects of different sizes and shapes with our prosthetic bionic hand. It can be easily found that our hands can adaptively grasp various objects.

In this part, we invited 10 volunteers to participate in these trials, including 9 able-bodied volunteers, aged between 28 and 36, and one volunteer with upper limb disability. Participants were all required to wear BIT hand C to grab different objects and move them from a table into a plastic basket. For each participant, they were asked to perform 6 trials for each object. Finally, we obtain grasping time data and success rate data from 60 trials for each object.

Volunteers were required to grasp a single object from initial status and then drop it in the basket. A trial was regarded a success when the objects were transported successfully without being dropped in this process. We defined the time this process spends as time-consuming. The results are shown in Table 2.

As the results show, our system performs better in grasping objects, no matter what shape and size it is, and the success rate can be all above 90%. Besides, people only need a little time to grasp the objects, almost no more than 5 s, which shows that our prosthetic hand has a better real-time performance. We can also conclude that with the size increase, the success rate can be improved, and the grasping time is shorter. This means that if the object is too small, the system should adjust their body posture repeatedly and then take more time for adaptive grasping. Therefore, the prosthetic bionic hand can meet the daily life application in a variety of tasks.

## 5. Conclusions

In this paper, we developed a humanoid prosthetic bionic, hand BIT hand C, which is controlled by two electrodes placed on the skin surface. This system can identify hand opening and grasping movements with a high accuracy of 96.59% based on our experiments. The hand can realize adaptive grasping through connecting rod mechanism. At the same time, two control loops, including the current loop and the position loop, are adopted in the control strategy to ensure the adaptive gripping of the artificial hand. As results show, our system performs well in grasping objects of different sizes and shapes, which has the potential to be applied for individuals with upper-limb differences. A thing to be noticed is that our prosthetic hand can only help restore some functionality to grasping tasks so far. It is now a proof of concept for a new type of prosthetic hand. However, this functional and reliable hand might improve prosthesis satisfaction and decrease the cognitive effort and visual attention required to control the device. In future work, we will further increase the function and availability of the hand and make it more accessible to people.

## Figures and Tables

**Table 1:** Recognition accuracy of different classifiers for 6 subjects (S1–S6).

Accuracy (%)	S1	S2	S3	S4	S5	S6	Average
NB	87.5	95.31	84.38	93.75	98.44	92.19	91.93
SVM	95.31	96.88	96.13	96.88	97.88	95.31	96.40
DT	87.94	92.19	91.38	93.75	98.44	90.63	92.39
KNN	86.94	92.19	86.39	92.19	98.44	92.19	91.39
LDA	94.31	97.31	97.13	97.43	96.92	96.45	96.59

**Table 2:** Grasping results of different objects with 10 participants.

Shape	Size	Success Rate	Time-Consuming(s)
Sphere	Small	90.74%	4.84
	Middle	96.30%	3.94
	Big	100.00%	4.08
Cube	Small	98.15%	3.91
	Middle	100.00%	3.91
	Big	100.00%	3.73
Torus	Small	96.30%	4.36
	Middle	98.15%	4.20
	Big	100.00%	4.21
Pentagonal Column	Small	92.59%	5.11
	Middle	98.15%	4.31
	Big	100.00%	4.33
Cylinder	Small	90.74%	5.08
	Middle	96.30%	4.06
	Big	100.00%	4.16

## Data Availability

The data presented in this study are available on request from the corresponding author.
